# Trends on deaths from acute pesticide poisoning in Mexico, 2000–2021

**DOI:** 10.1590/1980-549720240001

**Published:** 2024-01-19

**Authors:** Ma. Elena Moreno-Godínez, Eugenia Flores-Alfaro, Isela Parra-Rojas, Irma Martha Medina-Diaz, Aurora Elizabeth Rojas-García, Cristian Avilés-Ramírez, Gabriela Campos-Viguri, Marco Antonio Ramírez-Vargas

**Affiliations:** I Universidad Autónoma de Guerrero, Facultad de Ciencias Químico-Biológicas, Laboratorio de Toxicología y Salud Ambiental, Chilpancingo, México.; II Universidad Autónoma de Guerrero, Facultad de Ciencias Químico-Biológicas, Laboratorio de Epidemiología Clínica y Molecular, Chilpancingo, México.; III Universidad Autónoma de Guerrero, Facultad de Ciencias Químico-Biológicas, Laboratorio de Investigación en Obesidad y Diabetes, Chilpancingo, México.; IV Universidad Autónoma de Nayarit, Secretaría de Investigación y Posgrado, Laboratorio de Contaminación y Toxicología Ambiental, Nayarit, México.

**Keywords:** Trend, Mexico, Pesticides, Mortality rate, Poisoning, Tendências, México, Pesticidas, Taxa de mortalidade, Envenenamento por pesticidas

## Abstract

**Objetive:**

To provide a comprehensive analysis of mortality trends from acute pesticide poisoning in Mexico from 2000 through 2021.

**Methods:**

The governmental records of deaths from acute pesticide poisoning were used. The age-standardized years of life lost and aged-standardized mortality rates were estimated. Significant changes in trends of annual percentage change were identified using Joinpoint regression.

**Results:**

Between 2000 and 2021, mortality was primarily observed in individuals aged 15 to 19 years. Males were the most affected. Self-inflicted pesticide poisoning was the primary registered reason for death. The age-standardized mortality rate from acute pesticide poisoning was reduced from 2012 to 2021 (APC: -4.4; p=0.003).

**Conclusion:**

This report is the first study about the mortality rate from acute pesticide poisoning in Mexico. The results provided evidence to consider in developing laws to prevent acute pesticide poisoning.

## RESUMO

**Objetivo:**

Fornecer uma análise abrangente das tendências de mortalidade por envenenamento agudo por pesticidas no México de 2000 a 2021.

**Métodos:**

Foram usados os registros governamentais de mortes por envenenamento agudo por pesticidas. Foram estimados os anos de vida perdidos estandardizados por idade e as taxas de mortalidade estandardizados por idade. Modificações significativas nas tendências de variação percentual anual foram identificadas usando a regressão *Joinpoint*.

**Resultados:**

Entre 2000 e 2021, a mortalidade foi observada principalmente em indivíduos na faixa etária de 15 a 19 anos. Os homens foram os mais afetados. O envenenamento por pesticida autoinfligido foi o principal motivo de morte registrado. A taxa de mortalidade estandardizada por idade por intoxicação aguda por pesticidas foi reduzida de 2012 a 2021 (*Annual Percent Change* — APC: -4,4; p=0,003).

**Conclusão:**

Este relatório é o primeiro estudo sobre a taxa de mortalidade por intoxicação aguda por pesticidas no México. Os resultados forneceram evidências a serem consideradas no desenvolvimento de leis para prevenir o envenenamento agudo por pesticidas.

## INTRODUCTION

Occupational, para-occupational, and environmental exposure to pesticides has been associated with developing several human diseases and disrupting environmental health^1,2^. The growing need for generating and ensuring the production of agricultural products attributed to population growth is associated with increased pesticide use^3^. Furthermore, pesticides are employed to control vector-borne diseases (for example, Dengue, Zika, Chikungunya, and others), and the alarming insect resistance to pesticides is related to using new pesticide formulations characterized by high acute toxicity^4^.

Exposure to high doses of pesticides at short times results in an abrupt loss of homeostasis, leading to several physiopathological alterations. In the early 1970s, the World Health Organization (WHO) estimated 500 thousand cases of acute pesticide poisoning per year (WHO Expert Committee on Insecticides and Organization 1973) (this estimate only included accidental poisoning); in the 1980s, acute exposure to pesticides was recognized as a priority problem of public health by the WHO^5^. At the same time, most countries do not know the number of deaths from acute pesticide poisoning^6^.

A recent global study about the risk of pesticide pollution indicates that Mexico has a medium to high risk of pesticide pollution. This fact is related to the higher use rate of pesticides. Pesticide use is approximately 2-fold higher in Mexico than the world average^7^. According to the Food and Agriculture Organization Corporate Statistical Database (FAOSTAT), in the last decade (from 2010 to 2020), an average of 0.5 tons of pesticides were used per thousand hectares of land used for agricultural production in Mexico^8^. Agricultural production is associated with acute pesticide poisoning in Mexico; this public health problem was projected to increase with the advent of a technological package that includes new pesticide formulations under the North American Free Trade Agreement (NAFTA)^9^. This projection has been verified; the number of cases of acute pesticide poisoning is 4-fold higher after the application of NAFTA than in previous periods. According to a Mexican governmental report, the cases of acute pesticide poisoning have increased over time from 2000 to 2019. The national average of acute pesticide poisoning was 3,500 persons per year^10^. However, reports about mortality attributed to acute pesticide poisoning are unavailable. For this reason, this study aimed to perform an epidemiological description using the open data of the registered deaths by pesticide intoxication from the Information System of Health of Mexico. The data analyzed was from 2000 through 2021.

## METHODS

A retrospective descriptive study was carried out using the epidemiological database from “Secretaría de Salud” (Mexico’s Health Secretary); the records for 2000 through 2021 of deaths associated with acute pesticide poisoning in Mexico were considered. The registered cases included all patients with a recent history of pesticide exposure; pesticide exposure could be confirmed by anamnesis, physical examination, and laboratory test, according to “Sistema estadístico epidemiológico de las defunciones” (Epidemiological, statistical system of deaths)^11,12^.

The International Classification of Diseases and Related Health Problems, 10^th^ Revision (ICD-10)^13^ was used to identify cases. Code T60 indicates death associated with the toxic effects of pesticides. Therefore, the manner of death describes how a death occurs. The considered categories are accidental poisoning by pesticides (Code X48), intentional self-poisoning by exposure to pesticides (Code X68), assault by pesticides (Code X87), and pesticide poisoning of undetermined intent (Code Y18). Additionally, data extracted were restricted by age (five-year age groups), sex, federative state that registered the death, and the year of death registration were considered. The population was extracted from the National Institute of Geography and Statistics INEGI^14^ and National Population Council (CONAPO)^15^.

### Statistical analysis

We calculated the annual mortality rate per 100 thousand inhabitants ( $Annual\;mortality\;rate:\;\frac{No.\;of\;deaths\;from\;acute\;pesticide\;poisoning}{No.\;of\;persons\;in\;the\;population\text{​}at\;midyear}X\;100,00$ )^16^. Moreover, the age-standardized mortality rate per 100 thousand inhabitants ( $\sum_{i}\frac{d_{i}w_{i}}{y_{i}}$ ; *d_i_: No. of deaths; W_i_: Standard worl population according to WHO; Y_i_: person – years ar risk)*,^17^ and the age-standardized years of life lost rate per 100 thousand inhabitants $ASRY_{\left(c,s,t\right)}=\sum_{a}YLL_{rate(c,s,a,t)}\times W_{\left(a\right)};YLL_{rate}$ : years of life lost rate due to pesticide poisoning; c: pesticide poisoning; s: sex; a: age; t: period)^18^ were estimated.

Trends in the mortality parameters were assessed using Joinpoint regression (National Cancer Institute 2022). Joinpoint regression identifies the breakpoints (joinpoints) in which the linear trends are modified in direction or magnitude^19^. This method is performed using a log-linear model following a Poisson distribution $\text{log}(y_{i})\;=\;ℰ[y_{i}|x_{i}]\;+\varepsilon_{i}$ . The years from 2000 to 2021 are considered *X*_i_; the *Y*_i_ represents the annual rates; the *εi*. Represents residuals for the *í*th period, and the $ℰ[y_{i}|x_{i}]$ indicates the mean from regression. The mean is estimated considering a successive linear segment (n+1) over the period (from 2000 “a” to 2021 “b” ): $[a,\;b]:\;\varepsilon [y_{i}|x_{i}]=\beta_{0}+\beta_{1}x_{i}+\delta_{1}{\left(x_{i}-\tau_{1}\right)}^{+}+\cdots +\delta_{n}{\left(x_{i}-\tau_{n}\right)}^{+}$ . The annual percentage change (APC) describes the change in the constant percentage of the prior year’s rate. Moreover, this method indicates whether the modification on the trend line slope could be considered statistically significantly different from zero; we considered four maximum joinpoints and the 4,500 Monte Carlo permutations test. The joinpoint analysis was performed using the “Joinpoint Regression Program version 4.9.1.0”^20^.

## RESULTS

**Figura 1.**
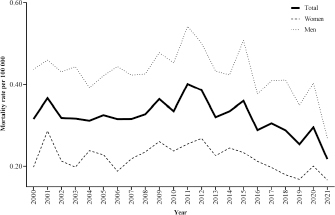
Mortality rate per 100 thousand inhabitants by acute pesticide poisoning in Mexico.

The mortality rates from acute pesticide poisoning per 100 thousand inhabitants in Mexico from 2000 to 2021 are presented in Figure 1. A total of 7,984 cases of deaths were attributed to acute pesticide poisoning. The highest mortality rate from total cases was observed in 2011, and the lowest rate was observed in 2021. Females’ mortality rates show that the lowest mortality rate was observed in 2021 and the highest in 2001. Males’ mortality rates showed the highest mortality rate in 2011. The mortality rate values for males were highest compared to females (Figure 1). A mortality rate (per 100 thousand inhabitants) from acute pesticide poisoning of 0.32 (confidence intervals — CIs 95% 0.27–0.39) was estimated for Mexico (from 2000 to 2021).

Joinpoint analyses for trends in the age-standardized mortality rate of registered deaths from pesticide poisoning in Mexico from 2000 to 2021 indicate significant variations in trends of annual percentage change (Table 1). From 2000 to 2012, an insignificant increase in the age-standardized mortality rate from acute pesticide poisoning was observed (Annual Percent Change — APC: 0.5; p=0.4). In contrast, a substantial reduction in the age-standardized mortality rate from acute pesticide poisoning was observed in the period from 2012 to 2021 (APC: -4.4; p=0.003). Similar results were observed for estimated trends by sex.

**Table 1.** Annual change in age standardized mortality rate from pesticide poisoning in Mexico (From 2000 to 2021).

Segment	Endpoint		APC (CI95%; p)		
	Lower	Upper	Total	Women	Men
1	2000	2012	0.5 (-0.8 1.9; 0.4)	0.7 (-1.1 2.6; 0.3)	1.4 (-0.1 3; 0.07)
2	2012	2021	-4.4 (-7 -3.5; 0.003)	-4.1 (-7.3 -0.8; 0.017)	-3.7 (-6.5 -0.7; 0.019)

APC: Annual percent change; CI: confidence interval.

**Figura 2.**
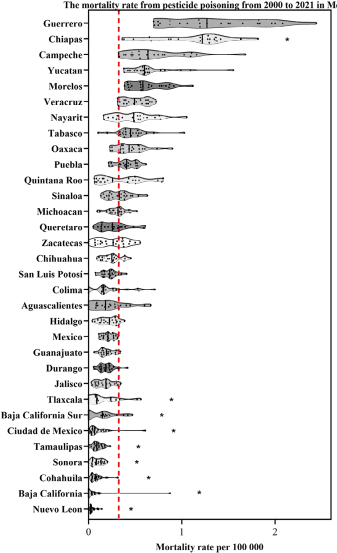
Mortality rate per 100 thousand inhabitants. Violin plots represent the mortality rate distribution per 100 thousand inhabitants for the state by studied years; the dotted red line indicates the national average for the analyzed period, *statistically significant difference compared with the national average (p<0.05).

The analysis concerning the national average versus federal states showed that Chiapas and Guerrero states had the highest mortality rate from acute pesticide poisoning compared to the national average. In contrast, the lowest mortality rate was observed for Baja California and Nuevo Leon states (Figure 2).

Intentionally self-poisoning by pesticide exposure was responsible for 55% of deaths. Moreover, thirty-two percent of deaths occurred by accidental poisoning (Supplementary Figure 1a). Men represented 65.2% of deaths from acute pesticide poisoning. The distribution of reasons for deaths from poisonings with pesticide by sex was as follows: intentionally self-inflicted poisoning >accidental poisoning>poisoning of undetermined origin>assault with pesticide (Supplementary Figure 1b). In Supplementary Table 1, the distribution of reasons for deaths from poisonings with pesticide are presented.

Considering the age group, the reasons for death from poisonings with pesticide is shown in Supplementary Figure 2. The age groups severely affected were 15 to 19 and 20 to 24 years. No intentionally self-inflicted poisoning was observed in age groups <9 years.

**Figura 3.**
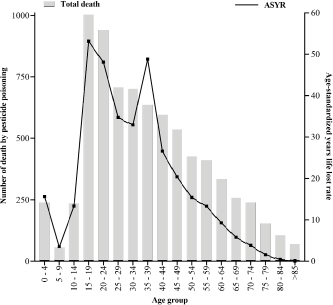
Age-standardized year of life lost rate per 100 thousand inhabitants. Mexico’s age-standardized years of life lost rate per 100 thousand from acute pesticide poisoning from 2000 to 2021 is represented.

A total of 330.4 age-standardized years of life lost (ASRY) rate per 100 thousand inhabitants were due to pesticide poisoning in Mexico (from 2000 to 2021). The ASRY associated with acute pesticide poisoning decreases steadily over age. Two peaks were observed in the 15 to 19 and 35 to 39 years. Eighty percent of ASRY linked to acute pesticide poisoning were observed between ten to 49 years (Figure 3). The trend of ASRY showed a significant reduction (APC: -4.71; p<0.001) from 2012–2021 (Table 2).

**Table 2.** Annual change in age-standardized years of life lost rate per 100 thousand inhabitants in Mexico (From 2000 to 2021).

Segment	Endpoint		APC (CI95%; p)
	Lower	Upper	
1	2000	2012	1.0 (-0.4 2.4; 0.2)
2	2012	2021	-4.7 (-6.7 -2.7; <0.001)

APC: Annual percent change; CI: confidence interval.

## DISCUSSION

Acute pesticide poisoning is recognized as a public health problem in the world. The use and abuse of pesticides have harsh consequences on human and environmental health. Exposure to the highest dosages of pesticides in a short time results in the abrupt loss of homeostasis, resulting in multiple pathophysiological alterations that could be causes of mortality. The present study analyzed the trends in mortality associated with acute pesticide poisoning in Mexico from 2000 to 2021. The results indicate that intentionally self-inflicted poisoning is the most common death from pesticide poisoning in Mexico.

Guerrero and Chiapas states showed the highest mortality rates from acute pesticide poisoning compared with the national average in the analyzed period (from 2000 to 2021). The agricultural practices in these states are characterized by production on a small scale. All family members generally work on their farming land, and the generated product is intended for auto-consumption or exchanging other products. Therefore, a lower percentage (32%) of producers operate in competitiveness, and generally, agricultural production is performed employing ancestral techniques that result in direct contact with pesticides^21-24^. Furthermore, the lowest levels of the human development index and healthcare access and quality index are associated with Guerrero and Chiapas states. Only Guerrero State has had the shortest life expectancies in Mexico^25^. This context could be associated with the highest mortality rate from pesticide poisonings observed in these states.

The increase in the number of attributed deaths to pesticide poisoning was observed in the present study. The same results have been observed in Costa Rica, Nicaragua^26^, and Brazil^27^. The use of highly hazardous pesticides is a commonly identified risk factor for acute pesticide poisoning^28^. For example, exposure to highly hazardous pesticides increased 2.77-fold the risk of death in Brazilians diagnosed with acute pesticide poisoning compared to exposure to pesticides with low /moderate/or high toxicity^29^. In this line, 46% of ingredient actives of pesticides registered and used in Mexico are considered highly hazardous pesticides according to Food and Drug Administration — FAO-WHO criteria^30^. Unfortunately, the records of death by acute pesticide poisoning in Mexico do not register the pesticide involved in the incident. Other variables, such as the rural or urban residence and the group chemical of the involved pesticides, are not available data. The lack of these data could be a real impediment to generating laws related to the regulation of the use of pesticides. More variables related to records are necessary for assessing the real impact of the use of pesticides on public health.

The public Mexican health system includes two sublevels of attention. The first level provides health care to formal workers from government and private organizations. Furthermore, the second level provides health care to the rest of the population without affiliation at the first level^31^. In 2011, the Mexican government consolidated the Health Information System (*Sistema de informacion en salud* — SIS:). The SIS increases the accuracy of records in national health statistics. This event could explain the peak in the registered mortality rate from 2011^32^. Another peak in the registered mortality rate was observed in 2015. In the same year, the importation of methamidophos, a highly hazardous pesticide, showed the maximum peak in the importation (1,620 tons) from 2010 to 2019^33^. Methamidophos is widely used in Mexico^34,35^. The increase in the accessibility to highly hazardous pesticides could be related to the mortality rate observed in 2015. Nevertheless, the information about death from acute pesticide poisoning can be considered the result of interaction between multifactorial factors resulting in complex repercussions.

According to WHO and FAO, self-inflicted pesticide poisoning is one of the most common methods of suicide in several countries. The use of highly hazardous pesticides is a similar risk factor observed in these countries^6^. Intentionally self-inflicted poisoning was the most frequent event in deaths from pesticide poisoning in Mexico (from 2000 to 2021). Similar results have been reported in Brazil^36^, India^37^, Malaysia^38^, China^39^, and South Korea^40,41^. In the mentioned countries, self-inflicted poisoning was the primary reason for death by pesticide poisoning. The mortality rate from suicide in Mexico has risen from 1990 to 2011; an increase of 250% was observed, and this event is most frequent in men compared with women^42^. The distribution of reasons for death from acute pesticide poisoning according to sex showed the same trends (intentionally self-inflicted poisoning>accidental poisoning>poisoning of undetermined origin>assault with pesticide); these data are contradictory with the previously reported other countries. For example, in China, suicide by pesticide poisoning was linked to females, and accidental pesticide poisoning was most frequent in males^43,44^; in Brazil, suicide by pesticide ingestion was common among men^29^. Trends in completed suicides in Mexico (from 1970 to 2007) showed that the use of a firearm (49%) and hanging (33%) were the main reported methods for suicide in men. In contrast, women select poisoning with drugs, pesticides, and lethal vapors as the primary method for suicide (59%)^45,46^. Nevertheless, this study showed that the principal cause of death from acute pesticide poisoning in females and males was attributed to suicide. More studies are necessary to establish the risk factors of suicide by pesticide intake.

The mortality rate from acute pesticide poisoning according to age group showed more predominantly in individuals between 15 and 19 years. This trend could be explained because the 15 to 19 years group is considered the most vulnerable group for performing suicidality in Mexico^42^. Furthermore, the mortality rate from acute pesticide poisoning decreases as age increases. These results contradict those observed in other countries such as China, Malaysia, South Korea, and Brazil, which reported an increase in mortality rate from pesticide poisoning as age increases. The authors suggested that the increase in mortality rate from acute pesticide poisoning is directly associated with self-intentionally poisoning by depressive psychological states^29,38,39,41^.

Furthermore, the analysis of suicide and suicidal behavior trends in Mexico from 1970 to 2007 showed that the highest levels of suicide and suicidal behavior were found in the 15–19 age group. An inverse relation was found with the age increase^46^. This observed conduct could explain the reduction in mortality rate from acute pesticide poisoning according to age increase.

The present article is the first study about the mortality rate from acute pesticide poisoning in Mexico. The results provided epidemiological evidence to consider in developing national laws to prevent acute pesticide poisoning. Also, this study emphasizes the need for accurate records of acute pesticide poisoning.

## SUPPLEMENTARY MATERIALS:

Supplementary Table 1
