# Supplementary material for: Trends on deaths from acute pesticide poisoning in Mexico, 2000–2021
Source: Rev Bras Epidemiol. 2024 Jan 19;27:e240001. doi: 10.1590/1980-549720240001 (PMC10798059; doi:10.1590/1980-549720240001)
Supplement: Supplementary file 1 [file 1980-5497-rbepid-27-e240001-Material-suplementar.pdf]

Supplementary Table 1.- Reported reasons for deaths by acute pesticide poisoning for State from 2000 to 2021

State (n)	Accidental poisoning with pesticides	Intentional self-poisoning by pesticide exposure	Assault with pesticide	Pesticide poisoning of undetermined intent
Aguascalientes (n=58)	25.9	72.2		1.9
Baja California (n=47)	89.1	6.5	2.2	2.2
Baja California Sur (n=19)	38.9	61.1		
Campeche (n=131)	17.6	71.2	0.8	10.4
Chiapas (n=1106)	51.9	42.1	2.3	3.7
Chihuahua (n=194)	48.3	45	0.6	6.1
CDMX (n=203)	34.8	55.1	1	9.1
Coahuila (n=52)	43.8	50	2.1	4.2
Colima (n=30)	37.9	58.6		3.4
Durango (n=72)	31.8	54.5		13.6
Guanajuato (n=240)	24.6	65.5	0.9	9.1
Guerrero (n=1023)	38.1	41.2	2	18.6

Supplementary Table 1.- Reported reasons for deaths by acute pesticide poisoning for State from 2000 to 2021

State (n)	Accidental poisoning with pesticides	Intentional self-poisoning by pesticide exposure	Assault with pesticide	Pesticide poisoning of undetermined intent
Hidalgo (n=129)	34.7	50		15.3
Jalisco (n=284)	18.6	75.5	0.4	5.6
Mexico (n=674)	12.7	73.5	8.9	5
Michoacan (n=285)	33.6	52.7	13.7	
Morelos (n=274)	25	57.2	1.7	16.1
Nayarit (n=120)	42.5	49.6		8
Nuevo Leon (n=40)	39.5	47.4	2.6	10.5
Oaxaca (n=376)	39.5	47.4	2.6	10.5
Puebla (n=508)	36	53		10.9
Queretaro (n=102)	27.3	66.7		6.1
Quintana Roo (n=93)	14	66		20
San Luis Potosí (n=131)	30	62		8.3
Sinaloa (n=195)	56	36	1.1	6.8

Supplementary Table 1.- Reported reasons for deaths by acute pesticide poisoning for State from 2000 to 2021

State (n)	Accidental poisoning with pesticides	Intentional self-poisoning by pesticide exposure	Assault with pesticide	Pesticide poisoning of undetermined intent
Sonora (n=51)	37	57		5.9
Tabasco (n=221)	25	73		1.8
Tamaulipas (n=67)	34	55		11
Tlaxcala (n=37)	35	54	2.7	8.1
Veracruz (n=823)	21	51	1.4	27
Yucatán (n=290)	14	82		4
Zacatecas (n=86)	38	54		7.6

n: number of deaths by acute pesticide poisoning. CDMX: Ciudad de Mexico. The data represented the estimated percentage for each reported cause of death by acute pesticide poisoning.
